# Lunacy in 1916

**Published:** 1918-02-23

**Authors:** 


### THE BOARD OF CONTROL.

### Lunacy in 1916.

The Report for the year 1916 of the Board of Control is recently issued, and contains more that is of general interest than these Reports usually contain.

The number of insane persons notified to the Board was on January 1, 1917, a few more than 134,000. This is a smaller number by more than 3,000 than those recorded on January 1, 1916, which, again, was fewer by more than 3,000 than the number recorded on January 1, 1915. For the second time within living memory, therefore, the number of the insane under care in England and Wales is diminished, and this diminution has extended over the last two years of the war. In the absence of the usual very elaborate statistical tables, which are, on account of the expense, no longer published, although they are prepared, we are unable to say authoritatively whether the diminution of numbers is wholly among the males, or whether, and in what degree, it is shared by the females; but as the proportion of males to females diminished during the last two years of record from 46.2 to 46 and then to 45.7, it should seem that this diminution in percentage is not enough to throw the whole weight upon the male side. The males appear to have lost 1,950 and the females 1,200 in the last year. If the decrease had been only among the males, it would have shown that a considerable number of men, who would in the ordinary course have become insane, had been drafted into the Army, and it may be surmised that the 2,000 cases of mental and nervous shock under observation in military hospitals on the date of this return included a considerable number of the 1,955 who escaped going into asylums by being drafted into the Army. The decrease in the number of insane women is not so easy to account for. Of 9,496 male attendants in asylums at the beginning of the war, 5,289 were of military age, and of these all have joined the Army except 950, yet in spite of the very great difficulty thus created in the administration of asylums, the number of suicides committed within asylum precincts is among the lowest on record, totalling for the year only four out of the 134,000 odd under care, as against an annual average of fourteen out of a very similar number. This result is, as the Report says, "especially creditable" to the asylum authorities, and "affords assurance that, notwithstanding difficulty, the large number of patients with strong suicidal tendency have been the objects generally of unremitting care and watchfulness." It is gratifying to discern in the Report of the Board of Control a more human note than characterised the Reports of the Commissioners in Lunacy. Such an expression of appreciation and approval as we have just quoted is quite foreign to the arid official record of the Lunacy Commission.

A novel legal incident is reported by the Board. At Bodmin Assizes two men pleaded guilty to indictments charging them with acts of gross indecency. They were bound over to come up for judgment if called upon, provided they agreed to go as voluntary boarders to two provincial licensed houses. The course taken by the judge seems to the Board of Control, and to us also, open to very grave objection. The provisions of the Lunacy Act, 1890, which authorise the admission of voluntary boarders into licensed houses and registered hospitals for lunatics, are based entirely on the voluntary action of the boarders themselves, and are intended to facilitate the early treatment of insanity while it is yet incipient; and it is expressly provided in the Act that the boarder shall be free to depart from the institution upon giving twenty-four hours' notice of his intention to do so. "We cannot think," say the Commissioners of the Board of Control, "it was ever intended to be applied to cases where [the Commissioners mean in which] consent to such residence was only given to enable a Court to relieve offenders of the consequences of their criminal acts. A consent so extorted is not, we would submit, such a voluntary act as was contemplated by the sections . . . and if adopted as a precedent, would be likely to bring the provisions of the sections into disrepute." We entirely agree. The voluntary boarder adopts that status in order to obtain early treatment of his malady, and at the same time escape being "stigmatised" as a lunatic. If the practice of this learned judge were to become a rule, the unfortunate boarder would escape being "stigmatised" as a lunatic only to be "stigmatised" as a criminal, and it is doubtful whether he would rejoice in the change.

The average weekly cost of maintaining pauper patients in county and borough asylums has risen from 10s. 9d. and 12s. 1½d. respectively in 1914-15, to 11s. 3¾d. and 12s. 10¼d. respectively in 1915-16. No doubt it will exhibit a further increase in the following years. Almost the whole of the increase is due to the enhanced prices of provisions. The total cost to the country of the service is £3,575,451, an enormous sum of money. The county and borough asylums are, as a rule, extremely well administered, and some superintendents, and those by no means the best, compete with one another in keeping down the cost of maintenance, a worthy object, no doubt, but one that is rather out of place as the primary object of a professional doctor is the treatment of the sick. We cannot but think that the unification of the Asylum Service and its administration as a department of State would be attended by the same advantages and the same economies as have been effected by the similar transfer of the prisons. One immediate benefit it would confer, viz.: that promotion of the members of the senior staff, and especially the medical staff, would be by merit, and not haphazard as at present. Any junior man who showed ability might then look forward with confidence to promotion to a higher post in some other asylum. At present he has no such confidence.

